# The role of authigenic sulfides in immobilization of potentially toxic metals in the Bagno Bory wetland, southern Poland

**DOI:** 10.1007/s11356-015-4728-8

**Published:** 2015-05-27

**Authors:** Beata Smieja-Król, Janusz Janeczek, Arkadiusz Bauerek, Ingunn H. Thorseth

**Affiliations:** Faculty of Earth Sciences, University of Silesia, Będzińska 60, 41-200 Sosnowiec, Poland; Department of Environmental Monitoring, Central Mining Institute, Plac Gwarków 1, 40-166 Katowice, Poland; Centre for Geobiology and Department of Earth Science, University of Bergen, Allégaten 41, 5007 Bergen, Norway

**Keywords:** Wetland, Metal sulfide, Biomineralization, Redox disequilibrium

## Abstract

The supply of Cd, Cu, Fe, Pb, Zn, and Tl into a wetland in the industrial area of Upper Silesia, southern Poland via atmospheric precipitation and dust deposition has been counterbalanced by the biogenic metal sulfide crystallization in microsites of the thin (<30 cm) peat layer, despite the overall oxidative conditions in the wetland. Disequilibrium of the redox reactions in the peat pore water (pH 5.4–6.2) caused by sulfate-reducing microorganisms has resulted in the localized decrease in Eh and subsequent precipitation of micron- and submicron-sized framboidal pyrite, spheroidal ZnS and (Zn,Cd)S, and galena as revealed by high-resolution scanning electron microscopy (SEM)/energy dispersive spectrometer (EDS). Saturation index for each sulfide is at a maximum within the calculated Eh range of −80 and −146 mV. Lead was also immobilized in galena deposited in fungal filaments, possibly at a higher Eh. Thallium (up to 3 mg kg^−1^) in the peat strongly correlates with Zn, whereas Cu (up to 55 mg kg^−1^) co-precipitated with Pb. The metal sulfides occur within microbial exudates, which protect them from oxidation and mechanical displacement. Vertical distribution of toxic metals in the peat layer reflects differences in pollution loads from atmospheric deposition, which has been much reduced recently.

**Electronic supplementary material:**

The online version of this article (doi:10.1007/s11356-015-4728-8) contains supplementary material, which is available to authorized users.

## Introduction

The precipitation of metal sulfides as a result of microbial sulfate reduction is one of the most important processes in aqueous metal reduction in constructed wetlands and other highly metal-contaminated organic-rich sediments (Johnson and Hallberg 2005; Batty et al. 2006; Sheoran and Sheoran 2006; Faulwetter et al. 2009; Lewis 2010; Yoon et al. 2012; Smieja-Król et al. 2014). Due to the low solubility of most sulfides, they provide a long-term sink for potentially toxic metals and keep the aqueous metal concentrations at environmentally permissible levels as long as the conditions remain anoxic (Kosolapov et al. 2004). There are many wetlands known to effectively remove toxic metals for a long time. For example, a natural wetland close to an abandoned lead-zinc mine in Ireland has been functioning unattended for over 120 years (Sheoran and Sheoran 2006). However, authigenic metal sulfides are not necessarily stored permanently, and wetlands constitute a danger of changing from net sinks to net sources of metals after a change of redox conditions to more oxic and subsequent sulfide oxidation (e.g., ElBishlawi et al. 2013; Johnston et al. 2014).

While there is a general agreement on the importance of sulfide mineral formation for metal containment in wetlands, the mineralogy of wetlands is rarely studied (i.e., Sonke et al. 2002; Kalaitzidis and Christanis 2003; Wüst and Bustin 2003; López-Buendía et al. 2007; Yoon et al. 2012; Cabała et al. 2013). The mineral content, their size, shape, chemical composition, and distribution in organic matrix are rather poorly known. The formation of metal sulfides is usually predicted from studying the changes in aqueous chemistry, e.g., the rates of metal removal during sulfate reduction/sulfide generation (Brennan and Lindsay 1996; Christensen et al. 1996; White and Gadd 1996; Webb et al. 1998; Batty et al. 2006; Stein et al. 2007; ElBishlawi et al. 2013), or their presence is inferred from sequential extraction procedures (Dellwig et al. 2002; Peltier et al. 2005) and from spectroscopic data (i.e., Peltier et al. 2003). Furthermore, the onset of conditions favorable for mineral formation and accumulation is still debatable for both natural and constructed wetlands (Gammons and Frandsen 2001; Kosolapov et al. 2004; ElBishlawi et al. 2013; Sochacki et al. 2014)

Zinc sulfide is the best studied metal sulfide in low-temperature, near-surface environments. Its nanocrystalline spheroidal aggregates, 1–5 μm in diameter, are composed of both ZnS polymorphs: sphalerite and wurtzite (Moreau et al. 2004). Zinc sulfide microspherules are important in controlling Zn and Cd distribution in Zn-rich peatlands (Smieja-Król et al. 2010, 2014; Yoon et al. 2012). Zinc sulfide microspherules were also confirmed to precipitate in a H_2_S-rich treatment wetland by Gammons and Frandsen (2001). Floccular aggregates of irresolvable submicron particles of (Zn, Fe)-sulfide were described from freshwater canal sediments in both urban and rural environments (Large et al. 2001).

Nanosized crystalline Pb and Cd sulfides have been synthesized in laboratory experiments using a variety of microorganisms as catalyzing agents (Dameron et al. 1989; Holmes et al. 1995; Ahmad et al. 2002; Gong et al. 2007). The precipitation of PbS and CdS is used in experimental designs to remove Pb and Cd from wastewaters (Velasco et al. 2008; Shpiner et al. 2009). Although similar precipitates are expected to form in nature, there are no detailed data on mineralogical features of such precipitates, despite their importance in predicting their short- and long-term stability.

In this paper, we describe the occurrence of micron- and submicron-sized metal sulfides (FeS_2_, PbS, ZnS, and (Cd,Zn)S) apparently precipitated due to the sulfate-reducing microorganisms (SRMs) activity in a thin (<30 cm) peat layer in a wetland developed in the abandoned sand mine in the eastern Upper Silesia urban-industrial region of southern Poland. High-resolution scanning electron microscopy (SEM) studies elucidating the microbial-mineral interactions are combined with bulk aqueous chemistry analyses conducted to follow the seasonal variations in metals and sulfate/sulfide concentrations.

## Study site

**Fig. 1:**
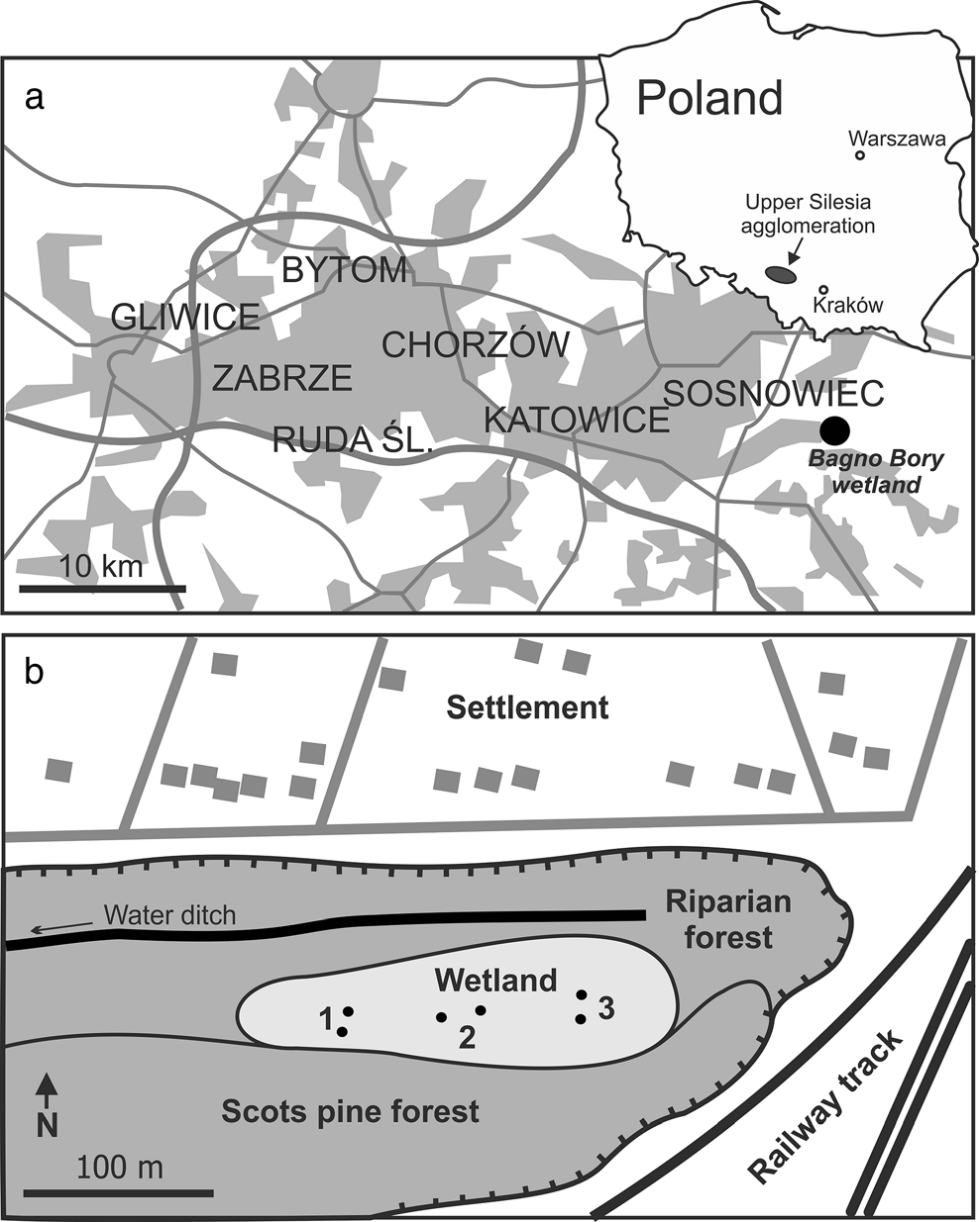
Location of the Bagno Bory wetland (**a**) in Upper Silesia agglomeration and (**b**) in the open pit. *Numbers* refer to the sampling sites

The Bagno Bory (BB) wetland (N 50° 16′ 34″, E 19° 16′ 20″, 270 m AMSL) covers an area of 2 ha (Fig. 1a). The wetland developed at the bottom of a shallow, water-logged open pit, left after sand mining prior to the Second World War (Chmura and Molenda 2007). The peat layer in the wetland is 15–30 cm thick and is deposited directly on fluvioglacial sandy and gravel sediments. Water percolates through the wetland westward. During our study, the water table was at or few centimeters above the peat surface in winter and spring and leveled out or remained up to 5–10 cm below the peat surface during summer.

The mean annual temperature for the wetland location is 8 °C. During winter (Dec.–Feb.), the average temperature is ca −1 °C and during summer (Jun.–Aug.) ca 18 °C. The mean annual precipitation between 1971 and 2000 was 729 mm (Ośródka et al. 2011). The mean annual potential evapotranspiration (PET) calculated by the Penman-Monteith method for the growing season (Apr.–Sep.) between 1970 and 2004 was 510 mm (Łabędzki et al. 2012). The maximum evapotranspiration occurs in July (Łabędzki et al. 2012). Details of the meteorological conditions for the wetland location during sampling period (2009–2011) are given in Smieja-Król and Bauerek (2015).

The Pleistocene fluvioglacials sands and gravels, 20–30 m thick, overlie the Carboniferous coal-bearing strata. Early Cretaceous Zn-Pb-Fe ore-bearing dolomites of the Mississippi Valley Type (MVT) deposits (Heijlen et al. 2003) crop out 3.5–10 km from the wetland. There is no hydraulic connection between the ore-bearing dolomites and the wetland.

Chmura and Molenda (2007) assigned the plant community *Drosera anglica—Oxycoccus palustris*, resembling transitional mire, to the BB wetland. Due to a variety of rare and endangered species (*Pinguicula vulgaris*, *D. anglica*, *Drosera rotundifolia*, *Liparis loeselii*), the BB wetland is protected within the Natura 2000 network under the European Union Habitats Directive (PLH240038).

Three sites were selected within the BB wetland for this study. They differ in plant species and in the water table level. Site 1 (Fig. 1b), in the western part of the wetland, is sphagnum-dominated (*Sphagnum denticulatum*) with addition of sundews (*Drosera anglica*, *Drosera rotundifolia*), cranberry marsh (*Oxycoccus palustris*), and common cotton grass (*Eriophorum angustifolium*) in the herbaceous layer. The vegetation of the eastern site (site 3) is dominated by common reed (*Phragmites australis*) indicating the highest water table level. The central site (site 2) consists of a dense herbaceous layer with the dominant white beak-sedge (*Rhynchospora alba*) in addition to plants vegetating site 1.

The BB wetland is located in an area significantly affected by past and present mining. The exploitation of the nearby Pb and Zn MVT deposits ceased in the 19th century. Material from old dumps has been locally used for road construction. Underground coal mining and sand mining in open pits continue in the region. The wetland is situated downwind the urban-industrial Upper Silesia agglomeration (Fig. 1a). As the result of atmospheric fallout, soils in the area are enriched in potentially toxic elements, including Pb (100–200 mg kg^−1^), Zn (400–800 mg kg^−1^), Cd (2–4 mg kg^−1^), and As (5–10 mg kg^−1^) (Lis and Pasieczna 1995).

## Material and methods

### Mineralogical and chemical analyses of solids

Three peat samples, each 10 × 10 × 2 cm in size, were collected in April 2009 from two depth intervals (5–7 and 15–17 cm) of the peat layer at each of the three wetland sites using a stainless steel knife.

The peat samples for SEM were stored at 4 °C for a few days, then fixed with 2 % glutaraldehyde for 1–2 h, dehydrated through a series of ethanol (EtOH) washes (15 min at 50, 75, 96, and 3 × 100 %), air-dried, mounted on aluminum specimen stubs, and carbon-coated. To control artifact formation, e.g., sample oxidation, critical point drying was used for comparison; still, it resulted in substantial loss of the material. Samples were analyzed using a Zeiss SUPRA 55VP field emission SEM equipped with a Thermo Noran System SIX energy dispersive spectrometer (EDS) system. Accelerating voltage of 4–15 kV and a working distance between 3 and 15 mm were used to satisfy conditions necessary for X-ray microanalyses, backscattered electron (BSE), and secondary electron (SE) imaging of individual peat components.

X-ray powder diffraction (XRD) was used for the identification of major mineral phases. Analyses were performed using the PHILIPS PW 3710 diffractometer (CoKα radiation) under the following operating conditions: voltage 45 kV, current 30 mA, impulse counting times 3, 8, and 9 s, and a step size of 0.01° 2θ. The phase identification was carried out using the X’ Pert plus software and the ICSD database (version 2007/12). Relative mineral abundances were determined semi-quantitatively by using ratios of the peak intensities of minerals.

Lead, Zn, Cd, Cu, Tl, and As concentrations were determined by Perkin Elmer ICP-MS spectrometer Elan DRC-e 6100, and Fe content was determined by atomic absorption spectrophotometry (AAS, Solaar M6) using air-dry material homogenized in a laboratory agate mill, re-dried at 105 °C, and burned at 460 °C for 24 h prior to digestion in concentrated HNO_3_ for 1 h. The quality of the analytical data for Pb, Zn, Cd, Cu, As, and Fe was checked with the reference material NIMT/UOE/FM/001 (acid-extractable concentrations, Yafa et al. 2004) with the measured values for Pb, Zn, Cd, Cu, and As falling within the acceptable limits of the reference material. The analytical accuracy for Fe was within ±15 %. The total carbon (TC) and total sulfur (TS) contents were determined using an Eltra Elemental Analyzer (model CS530). Calibration was made using Eltra standards. Analytical precision and accuracy were better than ±2 %.

### Sampling and chemical analyses of pore water

Pore water was sampled in early spring (March–April) and in summer (July) during 2010 and 2011 using 50 cm long and 5 cm wide plastic pipes, perforated at one side to allow water inflow from the bottom 15 cm of the peat layer. Two pipes (A and B, respectively) were inserted into the peat layer in each site (Fig. 1b). The pipes were acid washed and before each sampling, they were purged of stagnant water. Electrical conductivity (EC), pH, Eh, and dissolved oxygen were measured in the pipes using the integrated meter WTW MultiLine P4. Field Eh values were corrected with respect to the hydrogen electrode.

Pore water samples were collected in acid-washed polyethylene bottles and immediately stored in a cool box during transportation to a laboratory. The samples for metal analyses were filtered through 0.45 membrane filters and acidified to pH 2 by adding concentrated nitric acid (Suprapur, Merck, Germany). All water samples were then stored in a refrigerator until analysis was completed.

Concentrations of Ca, Mg, Na, K, Fe, and Mn were determined by AAS. Concentrations of Ba, Pb, As, Tl, Cd, and Zn were determined by inductively coupled plasma mass spectrometry (ICP-MS). Anions (Cl^−^, SO_4_^2−^) were determined using a Metrohm ion chromatograph after filtering the samples through 0.45 membrane filters the day after collection. All ion concentrations were corrected for procedural blanks, and accuracy was found to be within 5 % as checked against the SPS-SW2 Batch 120 Surface Waters Standard. Concentrations of sulfides were determined the next day after sampling by the thiomercurimetric method based on the titration with o-hydroxymercuribenzoic acid (HMB) in the presence of dithizone as an indicator and sodium hydroxide and disodium EDTA as a buffer to red color (Wronski 1971).

Dissolved organic carbon (DOC) was determined in un-acidified water samples filtered through 0.45 membrane filters using a Total Organic Carbon Analyzer (Shimadzu TOC 5000). DOC was calculated by subtracting inorganic carbon from total carbon.

The PHREEQC geochemical code (Parkhurst and Appelo 2013) with the included Minteq.v4 database was used to calculate the saturation indices (SIs) of the pore water in relation to selected mineral phases.

## Results

### Mineral inventory of the peat

The major minerals determined in the peat samples by XRD include, in decreasing order of abundance (Table S1): quartz, mullite, clay minerals (illite, kaolinite, smectite), feldspars, pyrite, and Fe oxides (hematite, maghemite/magnesioferrite). These were particularly abundant in the deeper (older) part of the peat layer (15–17 cm below peat surface) except for the site 3, in which both the sampling intervals (5–7 and 15–17 cm) were equally rich in minerals. This is further confirmed by the finding of almost the same ash content in both samples from the site 3 (Table S1).

Mullite is a high-temperature product of thermal decomposition of clay minerals during coal combustion (e.g., Vassilev and Vassileva 1996), and therefore, mullite is indicative of anthropogenic sources of dust particles deposited in the wetland.

Pyrite was detected by XRD exclusively in deeper parts of the peat layer in sites 2 and 3 (Table S1). Iron oxides occurred in similar quantities in all samples. The spherical shape of the numerous Fe oxide particles observed by SEM is typical of fly-ash particles abundant in the Upper Silesia (Jabłońska et al. 2003; Magiera et al. 2011). The strongly altered surface of the spheroidal Fe oxides suggests their limited stability in the wetland. Plentiful orange “fluffs” identified by SEM/EDS as Fe(hydro)oxides and Fe sulfates were observed on the wetland surface. Iron-(hydro)oxides commonly occurred as crusts on plant fragments and roots in the upper peat section.

Minerals that occurred below the detection limit of XRD, i.e., <1–2 wt%, but which were observed by SEM include galena (PbS), unspecified polymorphs of ZnS, and barite (BaSO_4_).

### Authigenic minerals

Authigenic minerals in the peat could be distinguished from the deposited atmospheric dust particles based on crystal morphology and the mode of their occurrence. The relative abundance of the authigenic minerals was estimated by simple counting of their individual grains observed under SEM. The authigenic minerals included, in decreasing concentrations: pyrite, galena, unspecified polymorphs of ZnS, (Cd,Zn)S, and, most likely, barite.

Pyrite occurred as framboids and aggregates of single octahedral crystals (Fig. S1). Pyrite framboids, ranging in diameter from 6 to 25 μm, were composed of discrete octahedral or, less frequently, cubo-octahedral crystals, 0.4 to 2.5 μm in size. The arrangement of crystals within framboids was mostly random; both loose and interpenetrant crystal packing was observed. The euhedral crystals and framboids were commonly embedded in extracellular polymeric substances (EPS) produced by microorganisms and colonized by bacterial cells (Fig. S1a). Pyrite crystals and framboids associated with microbial biofilms showed features indicative of dissolution, i.e., embayed grain boundaries, hollows, pitted and etched surfaces (Fig. S1b). They lacked signs of oxidation. The dissolution of the framboids proceeded from the outermost layer of crystals inwards, exposing deeper layers of crystal aggregates. The most decomposed regions of pyrite crystals were always in contact with the EPS slime (Fig. S1c).

**Fig. 2:**
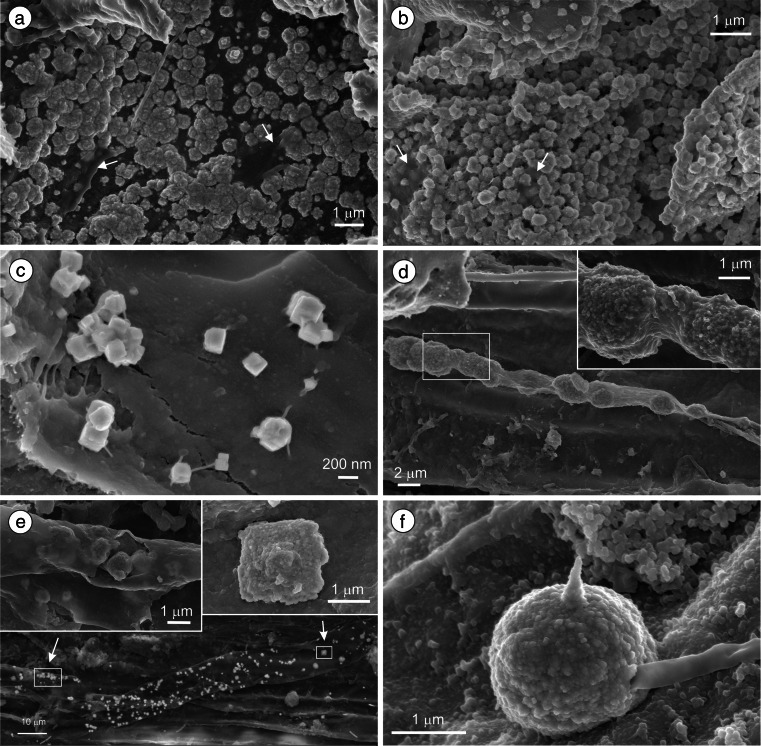
SEM images of authigenic PbS precipitates. **a** Crust and patches of PbS on a plant debris. EPS coverings are indicated by *arrows*. **b** Fine-grained irregular aggregates of PbS consolidated by microbial slime (*arrows*) and fibrils. **c** Galena cubes attached to a plant surface by microfibrils. **d** PbS microspheroids inside an organic filament, probably fungal hypha. **e** PbS associated with a fungal hypha. The *upper left inset* shows galena crystals inside the hypha wall; the *upper right inset* is an enlargement of cuboidal galena aggregate from the right part of the hypha. **f** Spherical PbS aggregate, probably a mineralized fungi spore

Galena was randomly distributed in the peat. It was locally abundant in deeper parts of the peat layer in sites 2 and 3. Galena commonly occurred as thin crusts or patches of precipitates, 0.15–0.6 μm in diameter, firmly attached to organic material (Fig. 2a). Less frequently, the precipitates were observed on inorganic surfaces, e.g., on framboids and within the voids of fractured fly ash particles. The roughness of the galena precipitates surface suggests that they were composed of nanometer-sized subindividuals. Those subindividuals aggregated into cuboidal or octahedral forms (Fig. 2a, e). Perfect cubes of galena no larger than 0.1–0.3 μm were observed occasionally (Fig. 2c).

Authigenic galena was commonly associated with microbial structures, in which individual PbS clumps ∼300 nm in diameter were consolidated by EPS biofilm (Fig. 2b). Some of the precipitates were associated with fungal hypha (Fig. 2d, e). Those precipitates were relatively large, up to 2.5 μm. Perfectly spherical PbS with roughened surface and diameter between 1.3 and 2.6 μm were most probably encrusted fungi spores (Fig. 2f).

Two morphological varieties of ZnS grains were observed in the peat samples. Angular grains larger than 1 μm with smooth surfaces are typical of atmospheric dust particles in Upper Silesia (Jabłońska 2003). Numerous hollows, pits, and fractures observed on their surfaces apparently developed after their deposition in the wetland.

**Fig. 3:**
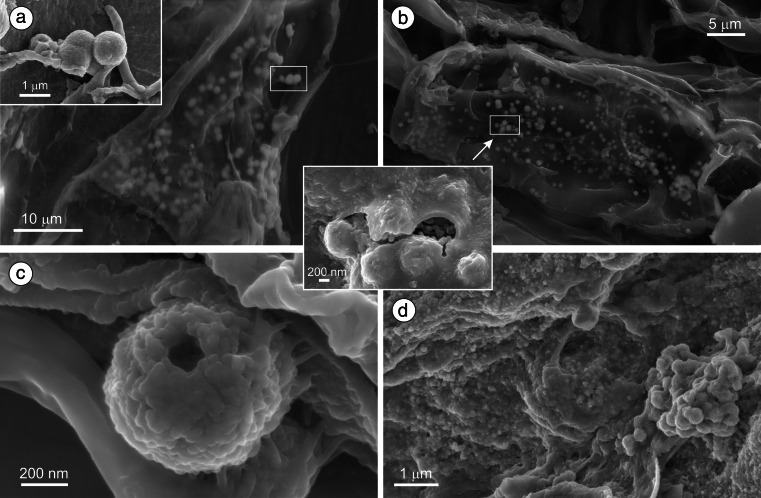
SEM images of authigenic ZnS. **a**, **b** Typical occurrence of ZnS spheroids enveloped by organic matter. The *upper left inset* shows uncovered ZnS spheroids attached to plant surface. The *inset in the center* is a close-up of spheroids inside the organic envelope. **c** Hollow ZnS aggregate attached to plant surface by microbial fibrils. **d** Tiny ZnS precipitates dispersed within a biofilm and an aggregate of ZnS spheroids

**Fig. 4:**
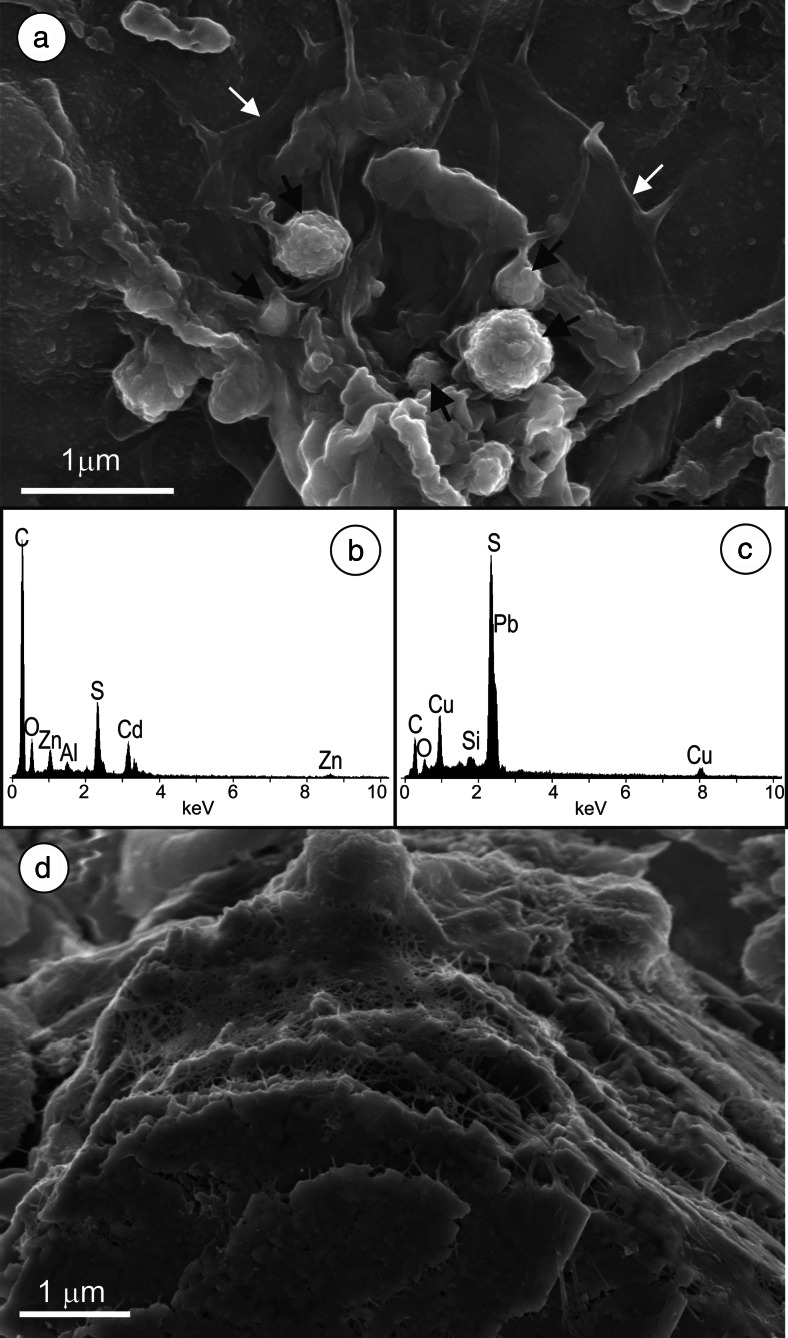
**a** Microspheroids of (Cd,Zn)S (*black arrows*) inside a collapsed EPS envelope (*white arrows*); **b** EDS spectra of the largest microspheroid from **a**; **c** EDS spectra of Cu-rich, galena particle (image not shown); **d** partially dissolved barite crystal covered by a dense network of microbial fibrils

The authigenic ZnS occurred as spheroidal aggregates of nanometer-sized particles, sometimes hollow (Fig. 3a, c). The microspheroids were between 0.3 and 1 μm in diameter, and over 50 % of them fell within the range of 0.5–0.7 μm (Fig. S2). Coarser ZnS spheroids were formed by coalescence or aggregation of microspheroids (Fig. 3d). A distinct feature of the ZnS microspheroids was their close association with peat organic matter. Most often, the microspheroids occurred deep inside plant cells and were always firmly adhered to the plant surface by microbial slime or microfibrils (Figs. 3a–c and 4a). Much smaller (50–100 nm) clumps of fine ZnS particles were observed within a mass of biofilms (Fig. 3d).

Some of the ZnS particles were enriched in Cd as revealed by SEM/EDS analyses. A few grains with Cd > Zn were observed (Fig. 4a, b).

The authigenic ZnS, (Zn,Cd)S, and PbS, similarly to pyrite, were common in the lower peat layer of site 2 and within both sampling intervals of the peat layer in site 3. However, those minerals rarely occurred together. They occupied different microsites.

Barite occurred as platy euhedral crystals loosely associated with organic matter. The barite plates were covered by a dense network of EPS nanofibrils and revealed different stages of decomposition (Fig. 4d). The barite crystal morphology is not sufficient to determine whether the barite originated in situ or whether it was deposited by atmospheric fallout. Euhedral platy crystals of barite are common constituent of atmospheric dust in Upper Silesia due to the Ba-enriched coal burning (Jabłońska et al. 2001). However, platy crystals of authigenic barite have been observed in another wetland in the outskirts of Upper Silesia (Smieja-Król et al. 2014).

### Trace elements in the peat

The peat layer contained elevated concentrations of Zn, Pb, Cd, and Tl (Table 1). The concentration of Tl (0.51–3.04 mg kg^−1^) was significantly higher compared to peat in unpolluted regions (0.01–0.7 mg kg^−1^; Dellwig et al. 2002). Arsenic, Pb, Zn, Cd, Cu, and Tl were mostly concentrated in the lower (i.e., older) part of the peat layer. This pattern of metal distribution within the peat layer reflects primarily differences in pollution loads from dust deposition, which has been much reduced recently. This is confirmed by the enrichment of mullite in the lower part of the peat layer. Unlike other metals, Fe was more abundant in the upper part of the peat layer in all three sites.

**Table 1:** Heavy metal concentrations together with total sulfur and carbon contents in the peat

	Site 1		Site 2		Site 3	
Depth (cm)	5–7	15–17	5–7	15–17	5–7	15–17
%						
TC	39.5	31.5	41	30.9	30.1	28.8
TS	0.59	2.11	0.96	1.49	2.77	2.93
mg kg^−1^						
Pb	293	634	201	890	596	595
Zn	107	2010	95.8	1370	560	4720
Cd	2.52	41.1	7.82	38.4	20.4	49.8
As	9.4	25.4	10.3	22.1	7.54	20.4
Cu	29.3	49.1	25.5	55.5	43.8	39.3
Tl	0.52	1.68	0.51	1.26	0.78	3.04
Fe	3800	3380	4430	2280	4100	3440

Each value is the mean of three parallel samples

The upper part of the peat layer in site 3 was enriched in Pb, Cd, and Cu relative to the two other sites. The content of Zn in both peat sampling intervals in site 3 was over two times higher than in sites 1 and 2 (Table 1).

Zinc concentrations were positively correlated with TS and negatively with TC (Table S2). This is in accordance with the SEM/EDS observations, implying that important pool of sulfur and zinc was bound in authigenic ZnS. There was a positive correlation between Cd and Zn (Table S2) as a result of the co-precipitation of both elements in (Zn,Cd)-sulfide (Fig. 4b). Cadmium concentrations as high as 4 wt% have been detected in ZnS in polluted mires north of the Upper Silesia agglomeration (Smieja-Król et al. 2010). Strong correlation between Zn and Tl (Table S2) suggests that Tl co-precipitated with Zn, however, in quantities too low to be detected by SEM/EDS in ZnS. Lead concentrations correlated positively with Cu and As (Table S2). The Pb and Cu co-occurrence in galena was confirmed by the EDS analysis (Fig. 4c). No linear relationship was observed between Pb and TS or TC.

### Peat pore water physicochemistry

#### Site-related variations

The most acidic pore water (average pH 5.4) and the lowest EC values (on average 255 μS cm^−1^) were observed in site 1 dominated by *Sphagnum* mosses. The highest pH (6.2) and EC (338 μS cm^−1^) values were measured in pore water of site 2, while at the third site, populated by the common reed, pH was 5.7 and EC was 325 μS cm^−1^. The DOC concentrations varied from 1.3 to 17 mg l^−1^, decreasing from site 1 (average 10 mg l^−1^) through site 2 (7.8 mg l^−1^) to site 3 (4.9 mg l^−1^). The concentrations of Ca and Mg in the pore water collected in site 1 were lower by a factor of 2 (4.9–18 mg Ca l^−1^ and 3.0–5.8 mg Mg l^−1^) in comparison with the two other sites (24–43 mg Ca l^−1^ and 5.4–9.9 mg Mg l^−1^). Site-related differences in trace element concentrations were negligible compared to the elements’ seasonal variations.

#### Seasonal variations

There was a significant difference in pore water chemistry between the two summer sampling campaigns in the three sites. Drought conditions and low water table in summer 2011 resulted in Eh, EC, and average concentrations of sulfates, Zn, Ba, Cd, and Tl an order of magnitude higher than those measured in the pore water sampled in summer 2010 (Table 2). The opposite effect was observed for dissolved sulfide, not detected in the oxidized pore water in summer 2011, while being found in concentrations of up to 11 mg l^−1^ in the pore water sampled in summer 2010. Concentrations of Cd exceeded Pb in the pore water from sites 1 and 2 in summer 2011. Despite similar sulfate concentrations and similar pH, EC, and Eh values in the pore water sampled in spring 2010 and 2011, metal concentrations were significantly different (Table 2). The decrease in metal concentrations was especially evident in summer 2010, relative to spring 2010. The low metal concentrations persisted till spring 2011. The concentrations of dissolved oxygen were Eh-independent and were higher in spring pore water. The As concentration increased with the decrease in redox potential (Table 2).

**Table 2:** Physical parameters (temperature (T), pH, Eh_m_, electrical conductivity (EC)) and chemical composition (dissolved organic carbon (DOC), dissolved oxygen, major anions, and metals) of peat pore water in the BB wetland

		Spring 2010 Mean (range)	Summer 2010 Mean (range)	Spring 2011 Mean (range)	Summer 2011 Mean (range)
T	°C	4.9 (4.1–5.7)	21 (18.2–24.6)	8.7 (6.3–15.7)	16.3 (15.4–17.3)
pH		5.6 (4.8–6.9)	5.8 (5.3–6.1)	5.7 (5.3–5.8)	5.1 (4.6–5.6)
EC	μS cm^−1^	307 (243–391)	361 (171–510)	264 (223–302)	588 (326–909)
Eh_m_	mV	166 (102–259)	11 (–47–69)	130 (56–293)	407 (292–509)
Oxygen	mg l^−1^	1.8 (1.5–2.5)	0.2 (0.1–0.4)	2.1 (1.7–2.5)	0.8 (0.05–1.5)
DOC	mg l^−1^	11 (6.3–17)	8.6 (4.6–14)	4.9 (1.3–8.6)	6.6 (4.1–10)
Anions					
Sulfate	mg l^−1^	77 (67–95)	43 (4.3–112)	76 (65–89)	141 (60–276)
Sulfide	mg l^−1^	n.a.	3.2 (0.2–11)	0.11 (0.08–0.20)	bdl
Chloride	mg l^−1^	20 (17–21)	25 (18–29)	24 (21–25)	8.8 (7.1–11)
Cations					
Na	mg l^−1^	9.3 (8.1–11)	11 (10–12)	n.a	n.a
K	mg l^−1^	2.9 (1.6–4.2)	3.1 (0.9–5.2)	n.a	n.a
Ca	mg l^−1^	31 (18–40)	26 (4.9–42)	n.a	n.a
Mg	mg l^−1^	7.6 (5.8–9.9)	6.1 (3.0–9.9)	n.a	n.a
Fe	mg l^−1^	7.5 (2.4–20)	7.7 (1.7–23)	n.a	n.a
Mn	mg l^−1^	0.9 (0.1–2.4)	6.1 (3.0–9.9)	n.a	n.a
Zn	μg l^−1^	176 (32–288)	44 (7.3–166)	23 (11–38)	633 (260–1290)
Ba	μg l^−1^	307 (104–738)	78 (32–188)	64 (41–129)	128 (49–334)
Pb	μg l^−1^	13 (2.2–33)	2.3 (0.4–9.3)	0.6 (0.3–1.6)	6.2 (1.3–15)
Cd	μg l^−1^	2.0 (0.2–5.1)	0.1 (0.01–0.4)	0.1 (0.1–0.2)	4.6 (1.6–9.8)
Tl	μg l^−1^	0.8 (0.3–1.1)	(bdl—0.03)	(bdl—0.1)	0.4 (0.1–0.6)
As	μg l^−1^	0.5 (0.2–0.8)	1.8 (0.8–4.4)	0.6 (0.5–0.7)	0.8 (0.6–1.2)

The mean (range) values of pore water from six pipes are given for each sampling campaign

*bdl* below detection limit: sulfide <0.04 mg l^−1^, Tl <0.01 μg l^−1^; *n.a.* not analyzed

### Saturation index

SI was calculated for the mineral species detected by SEM and/or XRD using both the measured Eh (Eh_m_) and the Eh calculated (Eh_S_) from aqueous sulfide/sulfate concentrations (Table S3). Sphalerite was taken for the evaluation of ZnS stability in the wetland, because sphalerite is expected to predominate over wurtzite and amorphous ZnS at low temperature and under biogenic conditions (Moreau et al. 2004). The stability of CdS was evaluated using thermodynamic data for greenockite.

The pore water was slightly supersaturated with respect to barite in most samples, with the SI values scattered close to the saturation level (Table S3). The pore water was occasionally slightly undersaturated with respect to hematite, in accordance with the observed progressive dissolution of spheroidal Fe oxides. The hematite saturation index was negative at site 1, regardless of the Eh value used in calculations, which is consistent with the lower pH at this site. Gypsum was unstable in the pore water as suggested by the negative SI values.

With the exception of one sample (1A in Table S3) collected in summer 2010, the pore water was highly undersaturated with respect to metal sulfides, if Eh_m_ was used in the geochemical calculations (Table S3). The lowest SI was obtained for pyrite, whereas sphalerite, galena, and greenockite had comparable levels of undersaturation. The lowest SI values with respect to all of the sulfides were obtained for the pore water sampled in summer 2011, which agrees with the aqueous sulfide content being below the detection limit (Table 2).

Application of Eh_S_ in the geochemical calculations results in the pore water being slightly supersaturated with respect to sulfides as indicated by positive SI values within a range of 3.3–4.3 for ZnS, 1.7–3.9 for PbS, and 1–3.5 for CdS. The saturation index was significantly higher for pyrite, ranging from 9.2 to 12 (Table S3).

## Discussion

The results demonstrate that potentially toxic metals (Zn, Pb, Cd, Cu, and Tl), originated in the wetland pore water from atmospheric deposition, were immobilized in a thin (<30 cm) peat layer through precipitation of sulfides. While calculations based on the S(II)/S(VI) redox couple suggest mildly reducing pore water in the peat layer, the high values of the measured Eh_m_ and the presence of dissolved oxygen (Table 2) are indicative of oxidative conditions unfavorable for crystallization of sulfides. The peat layer is too thin to maintain oxygen-depleted conditions characteristic for organic-rich environments, in which the decomposing organic matter serves as an electron source for reduction reactions.

To explain the precipitation of sulfides in the overall oxidative environment, we postulate a high degree of disequilibrium of the oxidation-reduction reactions in the peat pore water caused by SRMs. Their activity led to a localized decrease in Eh, supersaturation, and subsequent precipitation of metal sulfides in microsites. The presence of aqueous sulfides in the pore water (Table 2) provides indirect evidence for SRM activity because sulfide-producing inorganic reactions between dissolved sulfate and organic compounds are kinetically inhibited at low temperatures (Ohmoto and Lasaga 1982; Druschel et al. 2002).

The limited thickness of the peat layer makes the redox conditions sensitive to changes in temperature, precipitation, insolation, atmospheric pressure, and plant activity, sustaining the disequilibrium conditions. Fluctuations in the water table level during the summer cause aeration of the peat layer and consequently more intense oxidation of the reduced compounds. Bacterial growth and metabolic rates depend on temperature and are strongly reduced in winter. Bacterial sulfate reduction is reported to be approximately ten times higher in summer than in winter in a constructed wetland (Gammons and Frandsen 2001).

The mineral-forming reactants in wetlands are supplied mainly by atmospheric dust deposition and dissolution and by rainfalls and snow melting. Desorption from organic matter is an additional source of metals, especially during prolonged lower water table (Tipping et al. 2003). The bulk pore water concentrations of Zn, Pb, and Cd (Table 2) in the investigated wetland were low during the sampling period and generally reflected the lower limits of rainwater metal concentrations in Upper Silesia, with the exception of summer 2011, when concentrations of Zn and Cd in some pore water samples exceeded the concentrations of these metals in rainwater. The mean monthly rainwater concentrations of Zn, Pb, and Cd in 2010 in the center of the Upper Silesia agglomeration were in the range of 60–990, 2.4–19, and 0.15–6.5 μg l^−1^, respectively (Szymańska-Kubicka et al. 2011). The sulfate concentration in the peat pore water (Table 2) was generally an order of magnitude higher than in rainwater (1.7–11 mg l^−1^; Szymańska-Kubicka et al. 2011). The increased sulfate concentration relative to the rainwater may be explained by dissolution of both primary metal sulfides and gypsum particles deposited in the wetland. Gypsum is abundant in the deposited dust in Upper Silesia, particularly in winter (Jabłońska et al. 2003). Gypsum is unstable in the peat pore water (Table S3). The oxidation of reduced sulfur stored in organic matter is insignificant, as suggested by the similar sulfate concentrations in spring, i.e., during high water level, and in summer characterized by more pronounced water table fluctuations (Table 2). One would expect an accumulation of metals in pore water in response to the constant supply of metals from rainfalls and dust deposition, if there were no bacterially induced crystallization of metal sulfides in the peat layer.

In contrast to metals and sulfate ions, aqueous sulfide is probably less homogenously released to the pore water depending on the distribution of SRM. Microorganisms most likely colonized the peat unevenly, following the availability of easily degradable organic matter. Generally, monocots are susceptible to microbial degradation (Fabiańska and Kurkiewicz 2013), while sphagnum litter is decomposition-resistant (Hájek et al. 2011). This may explain the observed lower content of metal sulfides in the sphagnum-dominated site 1 in comparison with the two other sites.

**Fig. 5:**
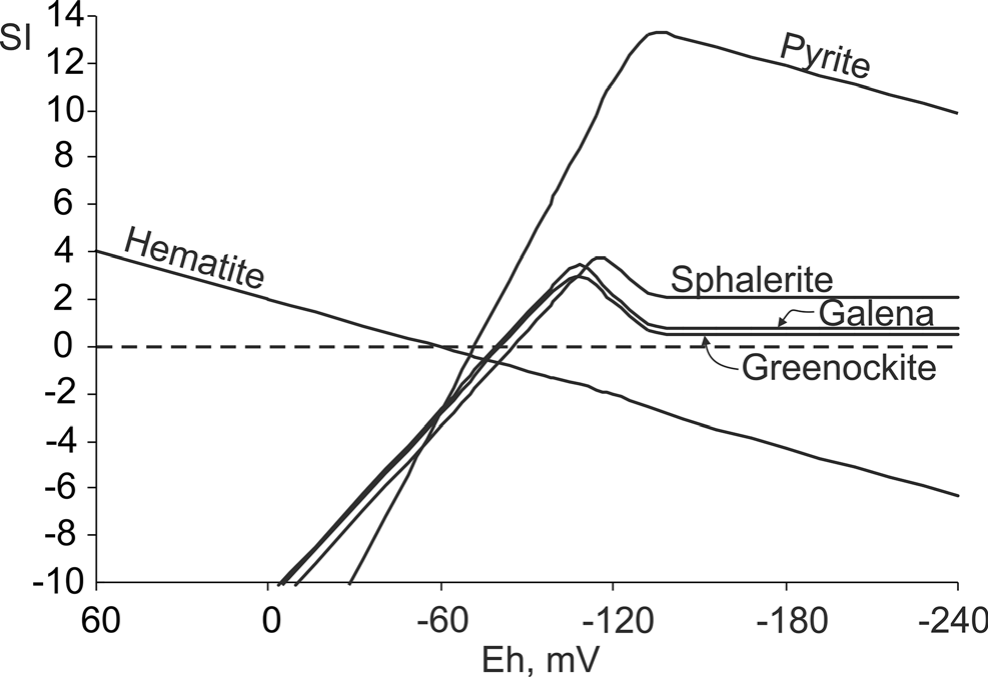
Saturation index for sulfides and hematite vs. Eh. Input data from sample 3A, summer 2010

Metal sulfides are highly sensitive to even small changes in Eh (Table S3 and Fig. 5). The limit of the supersaturation with respect to sulfides depends on the reactants’ concentration in the pore water (Fig. 5). The saturation index for pyrite reaches the maximum value within the −120 to −140 mV Eh range and decreases with the lowering of the redox potential (Fig. 5). This observation is in agreement with laboratory experiments, in which highly reduced solutions (−400 mV) were considerably less saturated with respect to pyrite than solutions with higher Eh and identical concentrations of Fe and S (Butler and Rickard 2000).

Similarly to pyrite, there is an optimum Eh range related to the highest SI for ZnS, PbS, and CdS. The maximum SI values for those minerals are obtained at a slightly higher redox potential (−100 to −120 mV) than pyrite. This observation suggests that Pb and Zn sulfides precipitated either slightly further away from the sulfide source than pyrite or their precipitation began shortly after the partial consumption of sulfide ions due to the pyrite crystallization. The latter possibility is supported by the SEM observations.

The calculated optimum Eh_S_ for the authigenic sulfides (Fig. 5) is within the range of −80 and −146 mV in the pore water (Table S3). Most likely, the Eh_S_ values were even lower in the microsites colonized by SRB in summer. During winter, with reduced bacterial activity, the Eh_S_ of the bulk pore water most probably equals that of the microsites.

The precipitation/dissolution of pyrite may control the dissolved sulfide concentration by acting as a buffer with respect to the other sulfides’ stability. Pyrite, due to its high sensitivity to redox changes, is the first sulfide to precipitate and the first one to dissolve in the wetland. The formation of framboidal pyrite requires high supersaturation, which translates into a high rate of pyrite nucleation (Ohfuji and Rickard 2005). Dissolution of pyrite (Figs S1b–c) may have been coupled to the precipitation of Fe(hydro)oxides at higher Eh. The conditions are expected to be more oxidized higher in the peat layer, especially during the summer’s lowered water table, inducing the movement and deposition of Fe in the upper peat layer. Iron is the only metal in the BB wetland that occurs in higher concentrations in the upper peat layer (Table 1). The attenuation of dissolved iron ions is necessary to allow the precipitation of Zn and Pb sulfides (Fig. 5). This might be achieved by pyrite precipitation and/or by the oxidation of Fe ions and subsequent precipitation of Fe(hydro)oxides along the vertical redox gradient.

The observed concentrations of dissolved Pb and Zn translate into comparable SI values, with the ZnS precipitation favoring slightly lower Eh (higher sulfide concentrations). The occurrence of PbS and ZnS in different microsites may have resulted from those small differences in Eh. The precipitation of pure Cd sulfide is limited or even inhibited due to the formation of ZnS–CdS solid solution.

The ZnS microspherules in the BB wetland are smaller (0.3–1 μm) than those described from an over 1-m-thick, Zn-polluted mire (1–3 μm; Smieja-Król et al. 2010) and those found in a flooded Pb-Zn mine (1–5 μm; Moreau et al. 2004). Perhaps, the smaller size of the microspherules resulted from lower Zn concentrations (<1.3 mg l^−1^) and the ephemeral conditions favorable for ZnS growth. The coarsening of ZnS aggregates occurred in the BB wetland through the attachment and aggregation of the individual microspheroids rather than the formation of concentric bands on the microspheroids by episodic precipitation and flocculation as observed by Moreau et al. (2004).

The size and morphology of PbS are much more variable compared to ZnS, with the largest PbS aggregates associated with fungal filaments (Fig. 2d–f). The association of PbS particles with fungi may have resulted from Pb sorption on fungal biomass. Excess of S(II) in the pore water led to the precipitation of the sorbed Pb as PbS. Fungi are known to be highly effective biosorbents. The metal binding by fungi is a passive process occurring on both living and dead organisms (Gadd 1990; Sterflinger 2000). An alternative explanation is provided by Ahmad et al. (2002). They showed in laboratory conditions that fungi, being predominantly aerobic organisms with high demands for aeration, are capable of precipitating CdS nanoparticles on their outer walls by secreting sulfate-reducing enzymes. The same or similar mechanism may have caused precipitation of PbS on the fungal filaments in the BB wetland.

## Conclusions

The constant supply of potentially toxic metals into the Bagno Bory wetland via atmospheric precipitation and dust deposition has been counterbalanced by the biogenic metal sulfide crystallization in microsites of the peat layer. Sulfate-reducing microorganisms play pivotal role in the precipitation and persistence of Zn, Cd, Fe, Pb sulfides in a thin peat layer by lowering redox potential in microsites in an otherwise oxidative environment. Additionally, Pb was also immobilized in galena deposited in fungal filaments, possibly even at elevated redox potential.

Cadmium and Tl co-precipitated with ZnS, while Cu was preferably immobilized in PbS. All of the investigated metal sulfides occurred within microbial exudates, apparently in the proximity of microorganisms that induced their crystallization. The microbial exudates protect sulfides from oxidation and mechanical displacement.

This study has shown that even a relatively thin layer of degradable organic matter is sufficient for metal sulfide crystallization and persistence, therefore, for their immobilization and retardation.

## Electronic supplementary material

Fig. S1 (DOC 366 kb)

Fig. S2 (DOC 64 kb)

Table S1 (DOC 38 kb)

Table S2 (DOC 33 kb)

Table S3 (DOC 58 kb)
