# Supplementary material for: The role of authigenic sulfides in immobilization of potentially toxic metals in the Bagno Bory wetland, southern Poland
Source: Environ Sci Pollut Res Int. 2015 May 27;22(20):15495–505. doi: 10.1007/s11356-015-4728-8 (PMC4620126; doi:10.1007/s11356-015-4728-8)
Supplement: Supplementary file 1 — (DOC 366 kb) [file 11356_2015_4728_MOESM1_ESM.doc]

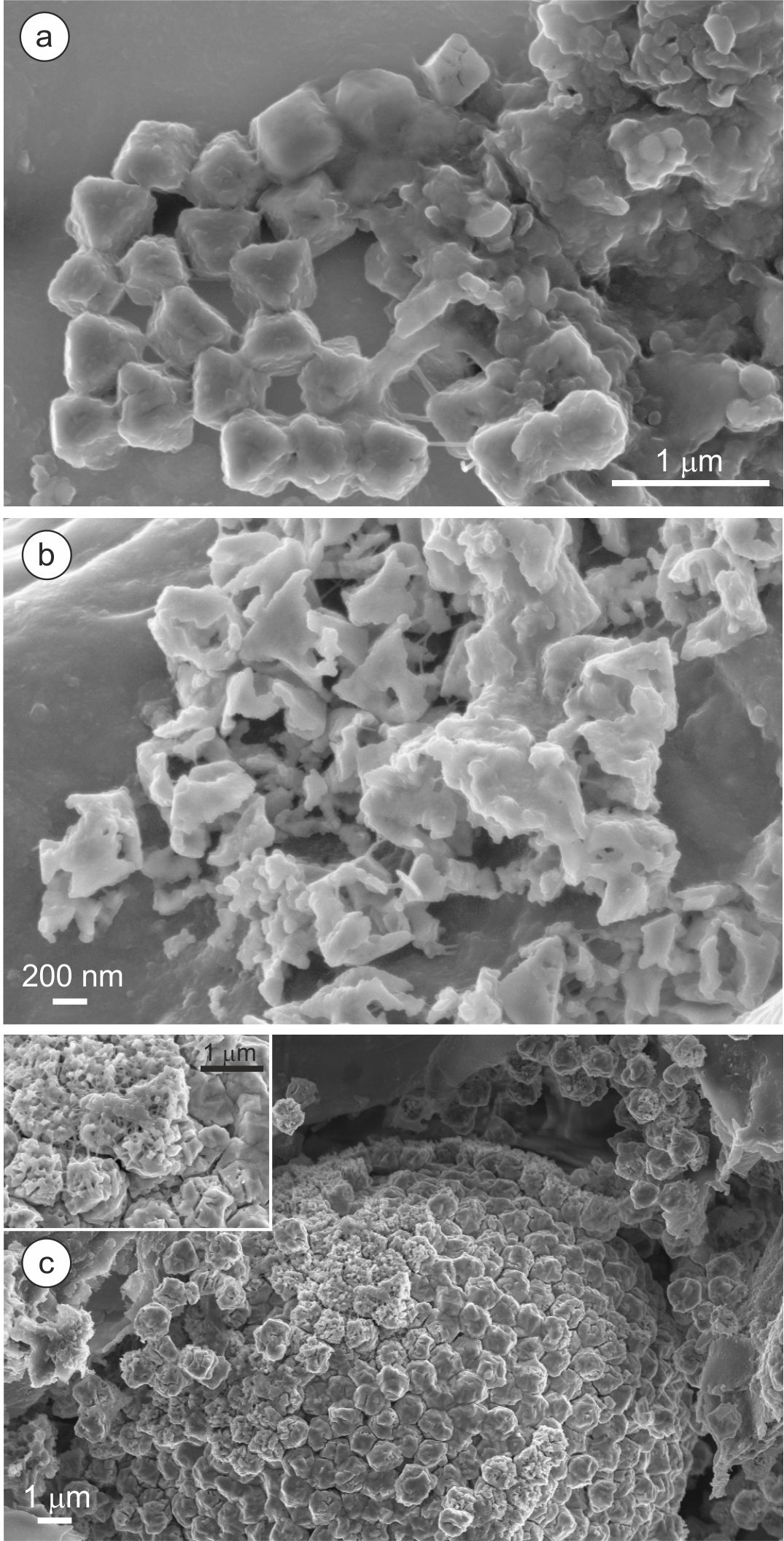


Fig. S1. SEM images of authigenic pyrite. (a) Octahedral crystals of pyrite embedded in microbial slime. (b) Highly dissolved pyrite crystals. (c) Decomposed framboidal pyrite with the outermost layer of crystals densely covered by EPS fibrils.
