# Supplementary figures and images for: The role of authigenic sulfides in immobilization of potentially toxic metals in the Bagno Bory wetland, southern Poland
Source: Environ Sci Pollut Res Int. 2015 May 27;22(20):15495–505. doi: 10.1007/s11356-015-4728-8 (PMC4620126; doi:10.1007/s11356-015-4728-8)

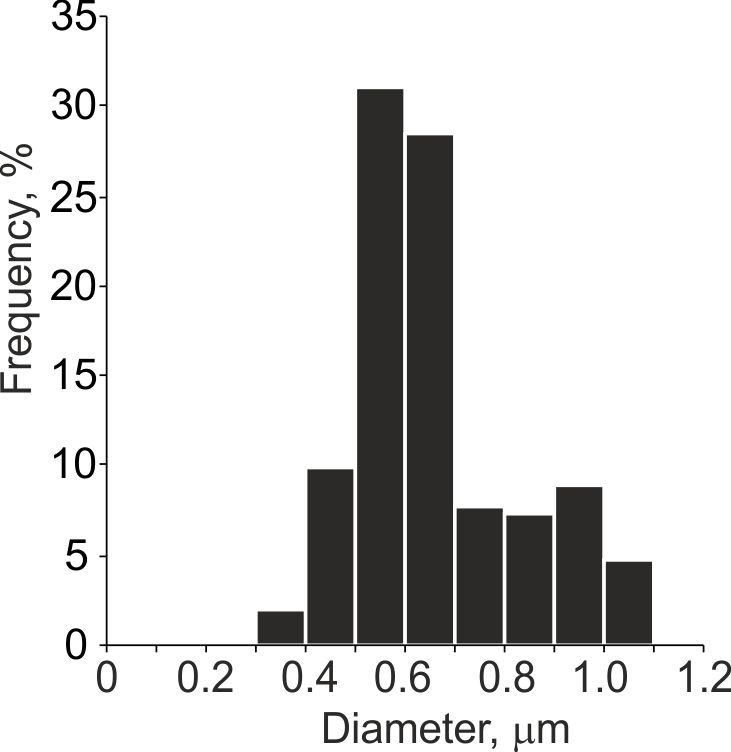


Fig. S2. Diameter distribution in ZnS spheroids.

Supplement: Supplementary file 2 — (DOC 64 kb) [file 11356_2015_4728_MOESM2_ESM.doc]
