# Supplementary material for: The role of authigenic sulfides in immobilization of potentially toxic metals in the Bagno Bory wetland, southern Poland
Source: Environ Sci Pollut Res Int. 2015 May 27;22(20):15495–505. doi: 10.1007/s11356-015-4728-8 (PMC4620126; doi:10.1007/s11356-015-4728-8)
Supplement: Supplementary file 3 — (DOC 38 kb) [file 11356_2015_4728_MOESM3_ESM.doc]

# Supplementary materials

**Table S1**. Relative proportions of minerals in peat samples determined from X-ray diffraction patterns and ash content.

|  | 1 site | | 2 site | | 3 site | |
| --- | --- | --- | --- | --- | --- | --- |
| Depth (cm) | 5-7 | 15-17 | 5-7 | 15-17 | 5-7 | 15-17 |
| Quartz | +++ | ++++ | +++ | ++++ | ++++ | ++++ |
| Mullite | ++ | ++++ | + | +++ | +++ | +++ |
| Feldspars | + | ++ | + | - | + | + |
| Clay minerals (illite, kaolinite, smectite) | + | ++ | - | +++ | + | + |
| Hematite | ++ | }++++* | ++ | }++++ | }+++ | }++++ |
| Pyrite | - |  | - |  |  |  |
| Maghemite/magnesioferrite | + | + | - | + | - | - |
| Gypsum | + | - | + | - | - | +++ |
| Halite | - | - | + | - | + | - |
| Ash (wt%) | 32 | 47 | 23 | 47 | 57 | 56 |

* in the presence of both hematite and pyrite, their relative proportions could not be determined due to overlapping of their major peaks and high background to peak ratio in the X-ray diffraction patterns.
