# Supplementary material for: The role of authigenic sulfides in immobilization of potentially toxic metals in the Bagno Bory wetland, southern Poland
Source: Environ Sci Pollut Res Int. 2015 May 27;22(20):15495–505. doi: 10.1007/s11356-015-4728-8 (PMC4620126; doi:10.1007/s11356-015-4728-8)
Supplement: Supplementary file 4 — (DOC 33 kb) [file 11356_2015_4728_MOESM4_ESM.doc]

**Table S2.** Correlation matrix for the trace elements, TS and TC contents in the peat.

|  | *As* | *Cu* | Cd | *Pb* | *Tl* | *Zn* | *Fe* | *TS* | *TC* |
| --- | --- | --- | --- | --- | --- | --- | --- | --- | --- |
| As | 1.00 |  |  |  |  |  |  |  |  |
| Cu | **0.64** | 1.00 |  |  |  |  |  |  |  |
| Cd | 0.49 | 0.20 | 1.00 |  |  |  |  |  |  |
| Pb | **0.65** | **0.89** | 0.32 | 1.00 |  |  |  |  |  |
| Tl | 0.33 | 0.08 | **0.68** | 0.06 | 1.00 |  |  |  |  |
| Zn | 0.40 | 0.01 | **0.75** | 0.02 | **0.91** | 1.00 |  |  |  |
| Fe | -0.06 | -0.13 | -0.17 | -0.32 | 0.07 | 0.04 | 1.00 |  |  |
| TS | 0.20 | 0.19 | **0.51** | 0.23 | **0.54** | **0.56** | -0.14 | 1.00 |  |
| TC | -0.37 | -0.26 | **-0.67** | -0.36 | -0.32 | **-0.55** | 0.33 | **-0.73** | 1.00 |
