# Supplementary material for: The role of authigenic sulfides in immobilization of potentially toxic metals in the Bagno Bory wetland, southern Poland
Source: Environ Sci Pollut Res Int. 2015 May 27;22(20):15495–505. doi: 10.1007/s11356-015-4728-8 (PMC4620126; doi:10.1007/s11356-015-4728-8)
Supplement: Supplementary file 5 — (DOC 58 kb) [file 11356_2015_4728_MOESM5_ESM.doc]

**Table S3**. Measured (Eh_m_) and calculated (Eh_S_) redox potential of pore water, and saturation index (SI) for relevant minerals in the BB wetland.

| Site/sample | Eh_m_  mV | Eh_S_  mV | SI_pyrite_ | SI_sphalerite_ | SI_galena_ | SI_greenockite_ | SI_barite_ | SI_hematite_ | SI_gypsum_ |
| --- | --- | --- | --- | --- | --- | --- | --- | --- | --- |
| Spring 2010 |  |  |  |  |  |  |  |  |  |
| 1A | 128 | - | -26 | -17 | -15 | -15 | 1.7 | -5.9 | -2.1 |
| 1B | 102 | - | -28 | -17 | -16 | -16 | 1.5 | -3.3 | -2.1 |
| 2A | 210 | - | -82 | -47 | -45 | -46 | 1.2 | 9.3 | -1.8 |
| 2B | 166 | - | -56 | -33 | -31 | -33 | 1.0 | 4.4 | -1.8 |
| 3A | 259 | - | -74 | -43 | -42 | -43 | 1.0 | 4.0 | -1.8 |
| 3B | 128 | - | -34 | -21 | -20 | -20 | 0.8 | -3.0 | -1.8 |
| Summer 2010 |  |  |  |  |  |  |  |  |  |
| 1A | -45 | -80 | 2.7 (11) | 0.4 (3.4) | 1.0 (1.7) | 0.4 (1.0) | 0.3 (0.3) | -5.2 (-6.4) | -2.8 (-2.8) |
| 1B | 32 | -85 | -16 (12) | -10 (3.7) | -9.3 (2.3) | -10 (1.6) | -0.1 (-0.1) | -1.1 (-5.1) | -3.3 (-3.3) |
| 2A | 69 | -130 | -37 (9.9) | -22 (3.6) | -21 (2.5) | -22 (1.9) | 0.3 (0.3) | 4.5 (-2.3) | -1.6 (-1.6) |
| 2B | -47 | -146 | -14 (9.4) | -8.2 (4.2) | -7.6 (2.8) | -8.9 (1.4) | -0.9 (-0.9) | 2.0 (-1.3) | -3.1 (-3.1) |
| 3A | 0 | -112 | -17 (10) | -11 (3.6) | -11 (2.7) | -11 (2.4) | 0.5 (0.5) | 2.0 (-1.9) | -1.8 (-1.8) |
| 3B | 60 | -118 | -34 (9.2) | -20 (4.2) | -19 (2.9) | -20 (2.5) | -0.2 (-0.2) | 2.1 (-4.0) | -2.4 (-2.4) |
| Spring 2011 |  |  |  |  |  |  |  |  |  |
| 1A | 56 | -52 | - | -12 (3.4) | -11 (3.9) | -12 (2.9) | 0.9 (0.9) | - | - |
| 1B | 104 | -85 | - | -23 (4.3) | -22 (3.7) | -23 (3.3) | 0.8 (0.8) | - | - |
| 2A | 107 | -74 | - | -23 (3.3) | -22 (3.5) | -22 (3.2) | 0.6 (0.6) | - | - |
| 2B | 293 | -95 | - | -50 (4.0) | -50 (3.3) | -50 (2.9) | 0.4 (0.4) | - | - |
| 3A | 112 | -83 | - | -24 (3.7) | -24 (3.4) | -24 (3.1) | 0.4 (0.4) | - | - |
| 3B | 113 | -76 | - | -23 (3.6) | -23 (3.7) | -23 (3.5) | 0.4 (0.4) | - | - |
| Summer 2011 |  |  |  |  |  |  |  |  |  |
| 1A | 392 | - | - | -60 | -60 | -59 | 0.9 | - | - |
| 1B | 378 | - | - | -58 | -57 | -55 | 0.9 | - | - |
| 2A | 403 | - | - | -51 | -60 | -59 | 0.8 | - | - |
| 2B | 467 | - | - | -64 | -64 | -62 | 1.7 | - | - |
| 3A | 292 | - | - | -38 | -37 | -37 | 0.8 | - | - |
| 3B | 509 | - | - | -40 | -38 | -38 | 0.3 | - | - |

Dashes – data not available,

In parenthesis – SI value calculated using Eh_S_
